# Perinatal stress and human hippocampal volume: Findings from typically developing young adults

**DOI:** 10.1038/s41598-018-23046-6

**Published:** 2018-03-16

**Authors:** Klára Marečková, Radek Mareček, Petra Bencurova, Jana Klánová, Ladislav Dušek, Milan Brázdil

**Affiliations:** 1 Behavioral and Social Neuroscience Research Group, Central European Institute of Technology, Masaryk University (CEITEC MU), Brno, Czech Republic; 2 Multi-modal and Functional Neuroimaging Research Group, Central European Institute of Technology, Masaryk University (CEITEC MU), Brno, Czech Republic; 3 Department of Neurology, St. Anne’s University Hospital and Faculty of Medicine, MU, Brno, Czech Republic; 4 Research Centre for Toxic Compounds in the Environment (RECETOX), Faculty of Science, MU, Brno, Czech Republic; 5 Institute of Biostatistics and Analyses (IBA), MU, Brno, Czech Republic

## Abstract

The main objective of this study was to investigate the impact of prenatal and early postnatal stress on hippocampal volume in young adulthood. In sharp contrast to numerous results in animal models, our data from a neuroimaging follow-up (n = 131) of a community-based birth cohort from the Czech Republic (European Longitudinal Study of Pregnancy and Childhood) showed that in typically developing young adults, hippocampal volume was not associated with birth weight, stressful life events during the prenatal or early postnatal period, or dysregulated mood and wellbeing in the mother during the early postnatal period. Interestingly, mother’s anxiety/co-dependence during the first weeks after birth did show long-lasting effects on the hippocampal volume in young adult offspring irrespective of sex. Further analyses revealed that these effects were subfield-specific; present in CA1, CA2/3, CA4, GC-DG, subiculum, molecular layer, and HATA, hippocampal subfields identified by translational research as most stress- and glucocorticoid-sensitive, but not in the remaining subfields. Our findings provide evidence that the type of early stress is critical when studying its effects on the human brain.

## Introduction

Early-life stress seems to have long-lasting effects on human health in later life including higher incidence of neurologic abnormalities, psychiatric disorders, age-related cognitive dysfunction, obesity and hypertension^1–7^. Animal studies suggest that early-life stress alters neurogenesis, programming of the hypothalamic-pituitary-adrenal (HPA) axis and neurobehavior^8,9^. Hippocampus, the key neural region for regulation of affect and HPA axis function^10^, is particularly sensitive to stress^11^. Animal research demonstrated that prolonged stress and increased glucocorticoids were associated with atrophy and retraction of the apical dendrites of the hippocampal pyramidal cells^12–14^ and disruption of neurogenesis in the hippocampus^15,16^. Both rats and tree shrews exposed to chronic stress exhibited reduced hippocampal volume^17,18^. In humans, psychological and psychosocial stress were related to altered structure of adult hippocampus^19–22^. Smaller hippocampal volume was described as a risk factor for development of stress-related psychopathology^23^ and a key neuroanatomical feature in anxiety disorder^24^. Smaller hippocampal volume was reported also in a number of other psychiatric disorders, including post-traumatic stress disorder, major depressive disorder, borderline personality disorder, schizophrenia, antisocial personality disorder, and dissociative identity disorder^25^.

According to Teicher and colleagues, sensitivity of hippocampus to stress is particularly heightened during the early developmental period^26^. The evidence is mixed in the literature, though. While adverse early life environment decreased neural stem cell production in juvenile mice^27^, separation of monkeys from their mothers was not associated with decrease of hippocampal volume^28–30^. Inconsistency is present also within human research. While recent research^31^ found smaller hippocampal volumes in youth and adults exposed to early life adversity, no similar relationship was found by a large population-based study from Australia^32^, a voxel-based morphometry study of neural correlates of prenatal stress in young women^33^, or a study relating maternal cortisol in pregnancy to hippocampal volume in childhood^34^. Relationship between early life events and hippocampal volume was also not found in a sample of women with remitted unipolar major depressive disorder^35^. Relationship between childhood abuse and smaller hippocampus was reported in depressed patients but not healthy controls^36^. One could hypothesize that the effects of early life stress on the hippocampal volume might be species-specific, sex-specific, and specific to the type and timing of the stress exposure.

Fetal sex modulates the responsiveness to prenatal stress^34,37–39^^,^ but as reviewed in MacQueen & Frodl, the effect of sex was not assessed in the majority of studies investigating the association between stress and hippocampal volumes^10^. For example, the three studies described above^33,35,36^ were done in females only. The low ability to find effects of early stress on hippocampal volume in female samples might be related to the protective effects of estrogen. Animal research showed differences in male and female rats in the effects of acute stress on performance in tasks involving hippocampal function, such as classical eye-blink conditioning or Y-maze or Morris Water Maze^40^. Sex differences were also found in the effects of maternal deprivation on development of synaptic plasticity in the hippocampus^41^. Moreover, estrogen treatment protected ovarictomized rats after chronic stress exposure from neuronal loss in the hippocampus^42^.

The main objective of this research was to study the effects of different types of prenatal and early postnatal stress on hippocampal volume in young adulthood and evaluate the potential modulatory role of sex. We focused on stress stemming from stressful life events during the prenatal or early postnatal period, low birth weight, which is known to be related to prenatal stress^43,44^, and symptoms of maternal postpartum depression including dysregulated mood and wellbeing, or anxiety and co-dependence.

As a secondary objective, we aimed to determine whether these effects might differ across the different hippocampal subfields. Most neuroimaging studies of early stress focused on potential abnormalities in the whole hippocampus, but the dorsal and ventral sectors of hippocampus have different functions^45^. Moreover, specialized function and different histological characteristics have been reported also in the different hippocampal subfields^46,47^. For example, the CA1 neuronal somata in the pyramidal layer have an ovoid shape and are populated sparsely, the CA2 neuronal somata are triangular in shape and larger than the somata of CA1, the CA3 neuronal somata are similar to those in CA2 but are more sparsely packed and the pyramidal layer is thicker than in CA2, and the somata in DG appear similar to those in CA3 but are more ovoid and sparse. Functionally, while CA1 serves for novelty detection and allocentric encoding^48,49^, CA3 and dentate gyrus serve for pattern separation and completition^50–52^. Thus, examining the effects of early stress on different hippocampal subfields may clarify the role of early stress on hippocampus and related behavior. Animal research showed that stress was related specifically to damage in the dentate gyrus, which contains multipotent adult neural stem cells and is thus the key region for neurogenesis^53,54^, and the CA3 subfield, which is the main target for glucocorticoids^55,56^. For example, rats subjected to physical restraint showed reduced branching and atrophy in CA3^57^ and tree shrews exposed to chronic psychosocial stress showed reduced dendritic length and number of branch points in CA3^14^. In humans, childhood maltreatment was associated with volume reductions in dentate gyrus, CA3 and subiculum^58^. Posttraumatic stress disorder (PTSD) was associated with smaller dentate gyrus and CA3 but not other hippocampal subfields, implying that PTSD is associated with selective volume loss^59^. Further research demonstrated that cortisol predicted volume of some (CA3, hippocampal head) but not other hippocampal subfields^60^. Whether exposure to prenatal or early postnatal stress might be associated with alteration of the same hippocampal subfields in humans is a critical but yet unanswered question that would clarify whether hippocampal subfields have unique sensitive periods when they are highly susceptible to the effects of early life stress.

## Results

### Prenatal stress and hippocampal volume in young adulthood

Means and standard deviations for the different measures of prenatal stress (birth weight, stressful life events in the first and second half of pregnancy) are provided in Table 1. There were no sex differences in any of these measures. There was also no relationship between the measures of prenatal stress and brain size corrected hippocampal volume (see all statistics in Table 2) or any interactions with sex. Correlations between the different measures of prenatal and early postnatal stress are presented in Supplementary Table 1.

**Table 1:** Means (M) and standard deviations (SD) for the different measures of prenatal and early postnatal stress (not log-transformed) by sex.

Measure of prenatal stress	Male offspring	Female offspring	Sex difference
	M, SD	M, SD	
Birth weight (g)	M = 3482.46, SD = 541.20	M = 3236.43, SD = 491.07	Not significant.
Stressful life events during first half of pregnancy	M = 0.25, SD = 0.27	M = 0.20, SD = 0.17	Not significant.
Stressful life events during second half of pregnancy	M = 0.17, SD = 0.13	M = 0.17, SD = 0.15	Not significant.
**Measure of early postnatal stress**	**M, SD**	**M, SD**	
Stressful life events during first six months after birth	M = 0.19, SD = 0.15	M = 0.17, SD = 0.13	Not significant.
Stressful life events during 6 to 18 months after birth	M = 0.27, SD = 0.22	M = 0.25, SD = 0.21	Not significant.
Anxiety and co-dependence during first weeks after birth	M = 0.25, SD = 0.27	M = 0.20, SD = 0.17	Not significant.
Dysregulated mood and wellbeing during first weeks after birth	M = 0.80, SD = 0.30	M = 0.79, SD = 0.34	Not significant.
Dysregulated mood and wellbeing at six months after birth	M = 0.80, SD = 0.34	M = 0.70, SD = 0.27	Not significant.
Dysregulated mood and wellbeing at 18 months after birth	M = 0.79, SD = 0.31	M = 0.79, SD = 0.31	Not significant.

**Table 2:** Prenatal and early postnatal stress and their relationships with hippocampal gray matter (GM) volume (corrected for brain size) in young adulthood.

Measure of prenatal stress	Sample size	Left hippocampus	Right hippocampus
Birth weight	126	beta = −0.12, p = 0.19	beta = −0.03, p = 0.71
Stressful life events during 1st half of pregnancy	93	beta = −0.11, p = 0.29	beta = −0.09, p = 0.41
Stressful life events during 2nd half of pregnancy	122	beta = −0.07, p = 0.44	beta = −0.08, p = 0.38
**Measure of early postnatal stress**		**Left hippocampus**	**Right hippocampus**
Stressful life events during first 6 months after birth	124	beta = −0.007, p = 0.94	beta = −0.08, p = 0.37
Stressful life events during 6–18 months after birth	117	beta = 0.003, p = 0.97	beta = 0.0006, p = 0.99
**Anxiety/co-dependence during first weeks after birth**	**122**	**beta** = **−0.25, p** = **0.006, R**^**2**^ = **0.06**	**beta** = **−0.24, p** = **0.007, R**^**2**^ = **0.06**
Dysregulated mood and wellbeing during first weeks after birth	119	beta = −0.02, p = 0.81	beta = −0.06, p = 0.54
Dysregulated mood and wellbeing at 6 months after birth	124	beta = −0.10, p = 0.27	beta = −0.07, p = 0.47
Dysregulated mood and wellbeing at 18 months after birth	117	beta = −0.07, p = 0.44	beta = −0.08, p = 0.37

### Early postnatal stress and hippocampal volume in young adulthood

Means and standard deviations for the different measures of early postnatal stress, namely stressful life events (measured during the first 6 months and between 6 and 18 months after birth), dysregulated mood and wellbeing (measured during the first weeks, at 6 months, and 18 months after birth), and anxiety and co-dependence (measured during the first weeks after birth) are provided in Table 1. There were no sex differences in any of these measures. There was also no relationship between stressful life events or dysregulated mood and wellbeing in the mother during the early postnatal period and hippocampal volume (see all statistics in Table 2) or any interactions with sex.

**Figure 1:**
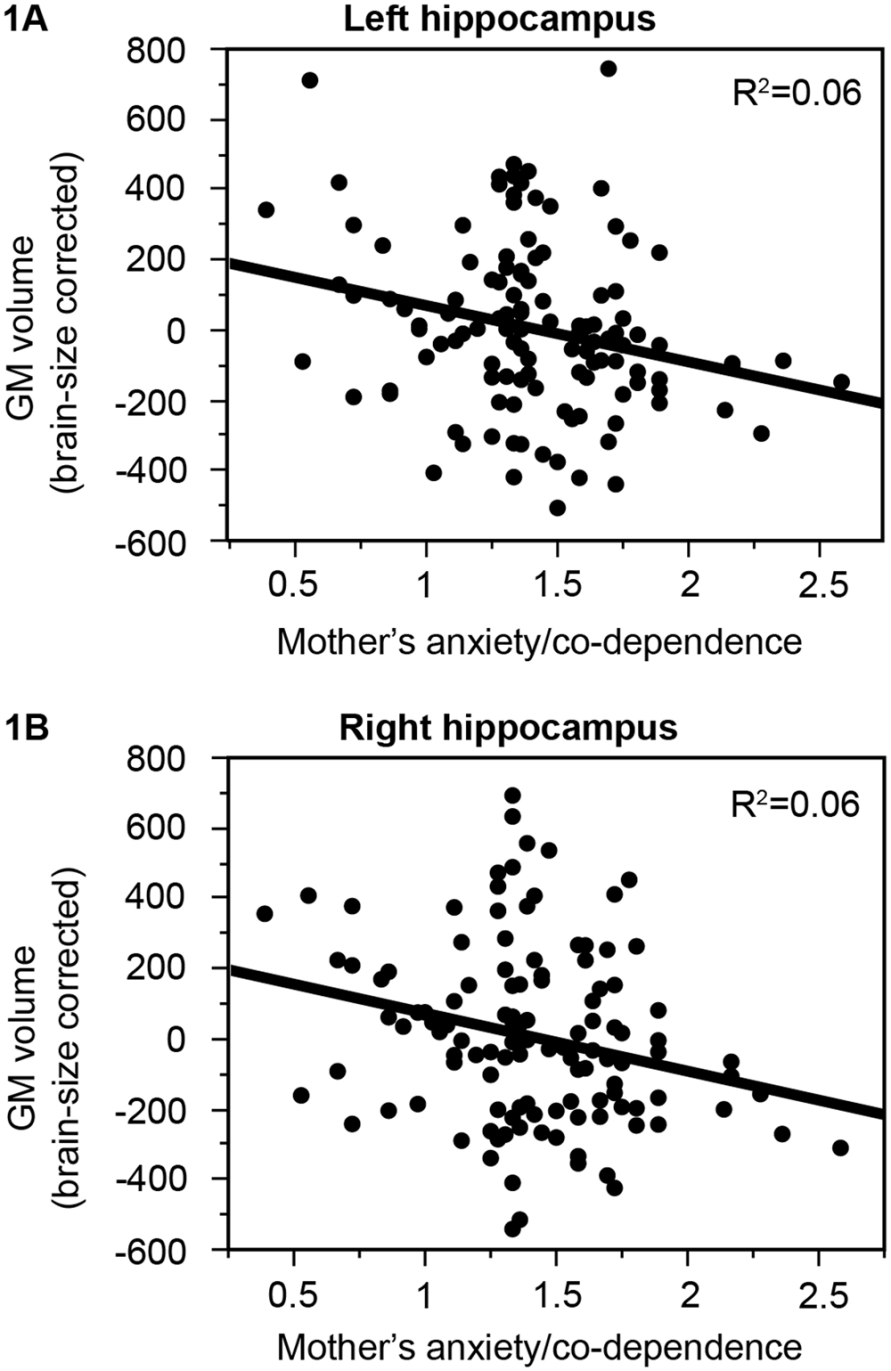
Mother’s anxiety/co-dependence and offspring’s hippocampal volume. Offspring of mothers with higher anxiety/co-dependence during the first weeks after birth had smaller gray matter volume of both left (**1A**; beta = −0.25, p = 0.006, R^2^ = 0.06) and right (**1B**; beta = −0.24, p = 0.007, R^2^ = 0.06) hippocampus (corrected for brain size).

Surprisingly, the offspring of mothers with higher levels of anxiety and co-dependence during the first weeks after birth had smaller volume of both left (beta = −0.25, p = 0.006, R^2 = ^0.06; Fig. 1A) and right (beta-0.24, p = 0.007, R^2 = ^0.06; Fig. 1B) hippocampus (brain size corrected). These relationships survived the correction for multiple comparisons (p < 0.008) and remained significant even when considering the potential modulatory role of the offspring’s sex. There were no interactions between mother’s anxiety/co-dependence during the first weeks after birth and the offspring’s sex (left hippocampus: F_(1,118)_ = 0.36, p = 0.55; right hippocampus: F_(1,118)_ = 0.86, p = 0.36). Additional multivariate regression analyses showed that the effect of Mother’s anxiety/co-dependence on hippocampal volume was independent of the other measures of early postnatal stress (left: beta = −0.20, p = 0.04; right: beta = −0.26, p = 0.009).

### Mother’s anxiety/co-dependence during the first weeks after birth and volume of hippocampal subfields in young adulthood

**Figure 2:**
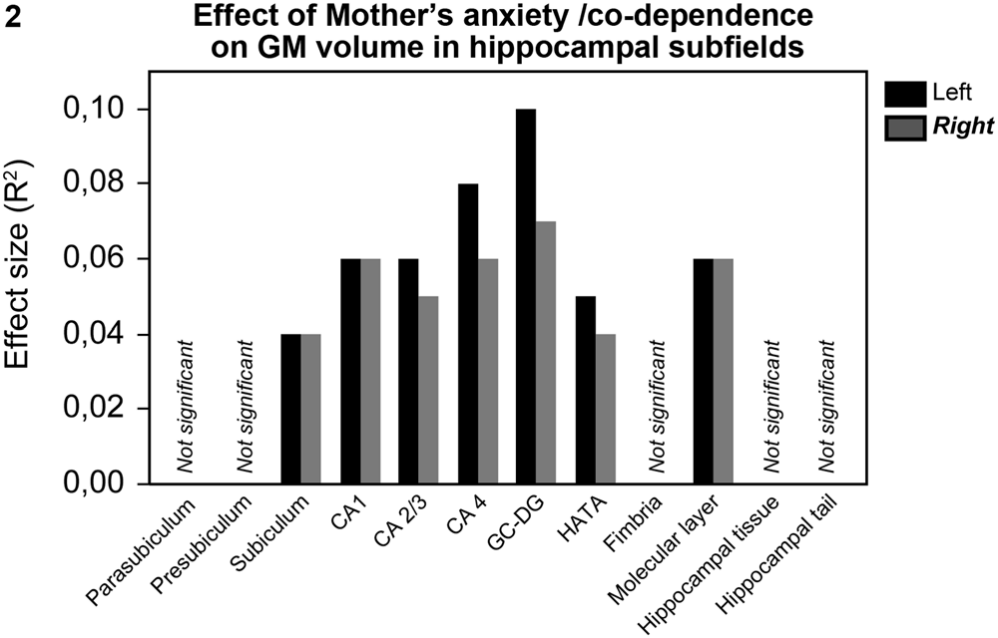
Effect of mother’s anxiety/co-dependence on offspring’s GM volume in hippocampal subfields. Offspring of mothers with higher anxiety/co-dependence during the first weeks after birth had smaller gray matter volume of left and right subiculum, CA1, CA2/3, CA4, GC—DG, molecular layer and HATA (corrected for brain size).

Further analyses in the 122 individuals followed up the relationship between mother’s anxiety/co-dependence during the first weeks after birth and offspring’s hippocampal volume, trying to determine whether the effects were subfield specific and thus affecting the volumes of hippocampal subfields to a different extent. Means and standard deviations for the volume of different hippocampal subfields are provided in Table 3. MANOVA showed significant interaction between mother’s anxiety/co-dependence and volume of the different subfields (corrected for brain size) in both left (F_(11,1320)_ = 3.63, p < 0.0001) and right (F_(11,1320)_ = 3.48, p < 0.0001) hippocampus. Posthoc analyses revealed that mother’s anxiety/co-dependence was associated with volume of left and right subiculum, CA1, CA2/3, CA4, GC—DG, molecular layer and HATA (see effect sizes in Fig. 2 and all statistics in Table 4). Left and right presubiculum, parasubiculum, hippocampal fissure, hippocampal tail andfimbria showed no relationship with mother’s anxiety/co-dependence during the first weeks after birth (see all statistics in Table 4). Again, none of these relationships were modulated by sex.

**Table 3:** Means (M) and standard deviations (SD) for the gray matter (GM) volume of different subfields in left and right hippocampus (not corrected for brain size).

Hippocampal subfield	GM volume (M, SD)	
	Left hippocampus	Right hippocampus
Parasubiculum	M = 71.51, SD = 13.24	M = 66.02, SD = 11.11
Presubiculum	M = 340.36, SD = 37.81	M = 314.20, SD = 35.54
Subiculum	M = 474.40, SD = 53.67	M = 457.19, SD = 49.02
CA1	M = 700.95, SD = 85.14	M = 720.85, SD = 87.57
CA2/3	M = 233.97, SD = 35.17	M = 246.94, SD = 37.08
CA4	M = 282.79, SD = 33.40	M = 288.54, SD = 34.11
GC-DG	M = 332.25, SD = 38.48	M = 336.51, SD = 39.27
HATA	M = 75.72, SD = 11.69	M = 71.87, SD = 10.34
Fimbria	M = 109.43, SD = 24.51	M = 107.37, SD = 21.64
Molecular layer	M = 630.57, SD = 67.62	M = 636.77, SD = 68.93
Hippocampal fissure	M = 151.8, SD = 23.56	M = 144.99, SD = 21.88
Hippocampal tail	M = 526.69, SD = 60.71	M = 539.27, SD = 71.53
Whole hippocampus	M = 3778.60, SD = 388.34	M = 3785.51, SD = 386.50

**Table 4:** Mother’s anxiety/co-dependence after birth and gray matter (GM) volume of hippocampal subfields (corrected for brain size).

Hippocampal subfield	Sample size	Left hippocampus	Right hippocampus
Parasubiculum	122	beta = 0.12, p = 0.20	beta = 0.11, p = 0.22
Presubiculum	122	beta = −0.0005, p = 0.99	beta = −0.04, p = 0.65
**Subiculum**	122	**beta **=** −0.20, p **=** 0.03, R**^**2**^ = **0.04**	**beta** = **−0.20, p** = **0.03, R**^**2**^ = **0.04**
**CA1**	122	**beta** = **−0.24, p** = **0.009, R**^**2**^ = **0.06**	**beta** = **−0.26, p** = **0.004, R**^**2**^ = **0.06**
**CA2/3**	122	**beta** = **−0.25, p** = **0.005, R**^**2**^ = **0.06**	**beta** = **−0.22, p** = **0.01, R**^**2**^ = **0.05**
**CA4**	122	**beta** = **−0.29, p** = **0.001, R**^**2**^ = **0.08**	**beta** = **−0.25, p** = **0.006, R**^**2**^ = **0.06**
**GC-DG**	122	**beta** = **−0.31, p** = **0.0004, R**^**2**^ = **0.10**	**beta** = **−0.27, p** = **0.003, R**^**2**^ = **0.07**
**HATA**	122	**beta** = **−0.21, p** = **0.02, R**^**2**^ = **0.05**	**beta** = **−0.19, p** = **0.04, R**^**2**^ = **0.04**
Fimbria	122	beta = −0.12, p = 0.20	beta = −0.05, p = 0.62
**Molecular layer**	122	**beta** = **−0.25, p** = **0.006, R**^**2**^ = **0.06**	**beta** = **−0.25, p** = **0.006, R**^**2**^ = **0.06**
Hippocampal fissure	122	beta = −0.03, p = 0.76	beta = −0.05, p = 0.57
Hippocampal tail	122	beta = −0.05, p = 0.60	beta = −0.05, p = 0.56

### Hippocampal volume and depressive symptomatology in young adulthood

Additional analyses showed that in our sample of typically developing individuals, there was no relationship between depressive symptomatology, as measured with Beck Depression Inventory (BDI), in young adulthood and brain size corrected hippocampal volume (left: beta = 0.11, p = 0.22; right: beta = 0.03, p = 0.72; n = 130) or between depressive symptomatology in young adulthood and mother’s anxiety/co-dependence during the first weeks after birth (beta = −0.11, p = 0.21; n = 122).

## Discussion

In this study, we have conducted a neuroimaging follow-up of a birth cohort to study the impact of prenatal and early postnatal stress on hippocampal volume in young adulthood. We showed that in typically developing humans, birth weight, stressful life events during prenatal or early postnatal period, and dysregulated mood and wellbeing in the mother during the early postnatal period were not associated with hippocampal volume in young adulthood and that sex did not modulate any of these relationships. Interestingly, mother’s anxiety/co-dependence during the first weeks after birth did show a relationship with volume of both left and right hippocampus in young adulthood and these results remained significant irrespective of sex. Further analyses revealed that the effects of mother’s anxiety/co-dependence during the first weeks after birth were subfield-specific; present in the hippocampal formation (CA1, CA2/3, CA4, GC-DG, subiculum), molecular layer and HATA but not in presubiculum, parasubiculum, fimbria, hippocampal fissure, or hippocampal tail.

Our results suggest that within typically developing individuals, mother’s anxiety/co-dependence during the first weeks after birth might be a much more sensitive predictor of hippocampal volume in young adulthood than the more objective measures of prenatal or early postnatal stress such as birth weight, number of stressful life events or the actual wellbeing of the mother. While the mechanisms are not clear, it might be that mother’s anxiety/co-dependence, characterized by great impact of others on her behavior, might result in her inconsistent behavior towards the infant, who then cannot detect the rules and remember the consequences of different actions, which might possibly represent much greater source of stress for the infant than, for example, a stressful life event in mother’s life.

The negative direction of the relationship between mother’s anxiety/co-dependence during the first weeks after birth and hippocampal volume in the offspring is consistent with the negative direction of the relationship between early stress and neurogenesis of the developing brain reported by animal research^61^. It is also consistent with others^62^ who showed that children of mothers with increased anxiety during the early postnatal period had smaller left hippocampal volume at 6 months of age. It was suggested these effects might reflect the transgenerational transmission of individual differences in vulnerability to anxiety-related disorders^62^. The subfield-specific results extend the findings of animal research showing evidence for the relationship between stress and alterations in CA subfields and dentate gyrus^63,64^ as well as research in humans reporting associations between childhood maltreatment and reduced volume of the CA3 subfield, dentate gyrus, and subiculum^58^. The lack of sex differences in the effects of early stress on hippocampal volume is in agreement with a review of the effects of prenatal stress on MRI outcome measures^65^ that showed that only one study so far reported sex differences in MRI outcome measures, namely the effect of higher cortisol at 15 weeks of gestation on larger volume of right amygdala in female but not male offspring^34^.

While the effect of mother’s anxiety/co-dependence was the same in left and right hippocampus (R^2^ = 0.06), the subfield-specific analyses showed a slightly larger effect size in the left (vs. right) CA2/3, CA4, GC-DG and HATA (see Table 4). This laterality is consistent with previous research^66^ showing that exposure to childhood maltreatment had the strongest associations with the volume of left CA4-DG and left CA2–3. The potential reasons why would the elevated levels of circulating corticosterone not affect both sides equally might include the differential distribution of NMDA and Glu receptor in the left vs. right hippocampus described by animal research^67^.

Previous studies reported small hippocampal volumes in at-risk adolescents, particularly those who experienced childhood adversity, already before the manifestation of clinical symptoms of major depressive disorder^68^. Both early-life adversity and smaller hippocampal volume were associated with a higher probability of depressive episodes during prospective follow-up^68^. In our sample of typically developing young adults, we did not observe any associations between depressive symptomatology, as measured with Beck Depression Inventory, and the brain size corrected hippocampal volume or between mother’s anxiety/co-dependence during the first weeks after birth and the brain size corrected hippocampal volume. While this absence of a relationship might be, in part, due to the relatively low variability in depressive symptomatology among the typically developing young adults, it is consistent with other structural studies that investigated the relations between depressive symptomatology and hippocampal volume and found no relationships^69–72^. Future research, designed as a prospective follow-up of our sample might try to determine whether individuals with smaller hippocampal volumes who were exposed to maternal anxiety/co-dependence after birth might develop depressive symptomatology in later life. Deficits in the function of hippocampus and the related negative affect network were characteristic for individuals with depressive symptomatology^10,73^. Recent research showed that reductions in hippocampal volume were apparent only in patients with an illness duration of at least 2.5 years or more than one episode of depression^74^.

Since hippocampal size is highly genetically determined^23,30^, further research should also clarify whether there might be any genetic modulators or mediators explaining our findings. For example, SNP rs7294919, which is known to have a particularly strong link to hippocampal volume^75,76^, might be a good candidate. Each copy of the T allele was associated with a 107.8 mm^3^ decrease in hippocampal volume^68^. The two neighbouring genes of rs7294919 are involved in apoptosis of neurons and hippocampal neuron dendrite growth, suggesting the possible mechanisms of action^68^. It might be that SNP rs7294919 might modulate the effects of mother’s anxiety/co-dependence on hippocampal volume.

The current study has a number of strengths. First, it was designed as a neuroimaging follow-up of a prenatal birth cohort, which means that our relatively large sample size consisted of individuals from a very similar background (all White Caucassians, typically developing, growing up in the same area and due to the early postcommunist era in Czechoslovakia in the early 90 s, they were born into families with very similar socioeconomical status) and a very narrow age range (23 or 24 years old), thus eliminating the effects of well documented age-related hippocampal atrophy^77,78^. Second, the unique data from European Longitudinal Study of Pregnancy and Childhood (ELSPAC-CZ) allowed us to draw on several approaches how to measure exposure to prenatal and early postnatal stress and evaluate their effects. Third, all measures of prenatal and early postnatal stress were based on data collected in the early 90 s, thus eliminating any possible false memories and recall bias. Fourth, we reported not only the effects on hippocampal volume but also the effects on individual hippocampal subfields, which are known to have different functions^47^. Fifth, the use of a software to measure the volume of hippocampus and its subfields allowed us to produce results that can be easily reproduced between laboratories.

The possible limitation of our study is the fact that the questionnaires assessing prenatal and early postnatal stress differ from the commonly used questionnaires on depression, social anxiety or co-dependence such as Beck Depression Inventory^79^ (BDI), Social Interaction Anxiety Scale^80^ (SIAS) or the Spann-Fischer Codependency Scale^81^ (SF CDS), however, the complete translation of the questionnaires is provided in the Supplementary methods. Another limitation is the fact that we cannot control for earlier/later maternal anxiety/co-dependence and thus determine whether the effects of mother’s anxiety/co-dependence are specific to early postnatal experiences. We hypothesize that while symptoms of mother’s anxiety/co-dependence might, in part, characterize mother’s behavior also prenatally and later in life, their effects on the offspring’s hippocampus would be particularly pronounced during the sensitive period of first weeks after birth. It is also important to note that while our previous work^82^ with this sample demonstrated an effect of prenatal stressful life events on the brain and particularly three brain regions known to be hypometabolic in depressed patients vs. healthy controls (i.e. mid-dorsolateral frontal cortex, anterior cingulate cortex, and precuneus), the birth weight of our typically developing participants was in the normal range (M = 3346.85, SD = 526.62; viz Table 1) and this might have limited the possibility to show an effect of birth weight on hippocampal volume.

We conclude that in sharp contrast to numerous animal models, within typically developing humans, birth weight, stressful life events during prenatal or early postnatal period, and dysregulated mood and wellbeing in the mother during the early postnatal period were not associated with hippocampal volume in young adulthood. But, mother’s anxiety and co-dependence during the first weeks after birth did show long-lasting effects on the hippocampal volume in the offspring irrespective of sex and these effects were most pronounced in the hippocampal subfields identified by translational research as most stress- and glucocorticoid-sensitive^63,64^. Our findings provide evidence that the type of early stress is critical when studying its effects on the human brain, possibly explaining the inconsistency in the literature.

## Methods

### Participants

Typically developing young adults from the European Longitudinal Study of Pregnancy and Childhood, the Czech Republic^83,84^ (ELSPAC–CZ), a prenatal cohort from Czech Republic whose members were born between 1991 and 1992, were invited to participate in a neuroimaging study *Biomarkers and underlying mechanisms of vulnerability to depression* (VULDE; FP7-IEF-2013) at Central European Institute of Technology, Masaryk University. A total of 131 individuals (61 males, 70 females) completed the neuroimaging protocol in 2015. All of the participants were 23 or 24 years old and of White Caucasian background. Ethical approval for the VULDE study was obtained from ELSPAC Ethics Committee and written informed consent was obtained from all the participants, including the agreement to merge data from VULDE with their historic data from ELSPAC-CZ. The methods of the study described bellow were in accordance with the relevant guidelines and regulations.

### Measures of prenatal and early postnatal stress

Between 1990 and 1994, mothers of our participants answered number of questionnaires regarding possible indicators of prenatal and early postnatal stress including stressful life events, dysregulated mood and wellbeing, and anxiety/co-dependence and provided information about the birth weight of the offspring (data available for 126 out of the 131 participants). The questionnaire about stressful life events was filled in at four time points to reflect stressful life events during the first (completed by 93 out of the 131 mothers) and second (completed by 122 out of the 131 mothers) half of pregnancy, during the first 6 months after birth (completed by 124 out of the 131 mothers), and during 6–18 months after birth (completed by 117 out of the 131 mothers). It consisted of 40 questions on stressful events such as break up or divorce with the partner, consideration of abortion, violence, serious illness or death in the family or financial difficulties answered by a 5-point Likert scale. The questionnaire on dysregulated mood and wellbeing was filled in at three time points, namely at first weeks after birth (completed by 120 out of the 131 mothers), at 6 months after birth (completed by 124 out of the 131 mothers), and at 18 months after birth (completed by 117 out of the 131 mothers) to reflect dysregulated mood and wellbeing in the past month. This questionnaire included 33 questions on anger, panic, sadness, fatigue, digestive problems, appetite, sleep and self-harming thoughts answered by a 4-point Likert scale. The questionnaire on Anxiety/co-dependence was filled in only during the first weeks after birth and completed by 122 out of the 131 mothers. It included 36 questions on anxious behavior and co-dependence such as worried about other people’s opinions, change behavior in order to please others, insecure when meeting new people, worried to say what she thinks because others might not like her, worried to be criticized, anxious when saying good bye, worried to lose a friend, all answered on a 4-point Likert scale. See the full list of questions in the three different questionnaires in Supplementary Methods.

### MRI Acquisition

In 2015, all participants were scanned using a 3 T Siemens Prisma MRI scanner. T1-weighted MPRAGE images of the whole brain were acquired with 64 channel head/neck coil using the following acquisition parameters: voxel size 1 × 1 × 1 mm, repetition time (TR) 2300 ms, echo time (TE) 2.34 ms, inversion time (TI) 900 ms, flip angle 8 degrees.

### Analyses

T1-weighted data were processed using Freesurfer version 6.0^85^ and the volume of total left and total right hippocampus was calculated. Volume of the different hippocampal subfields (left and right parasubiculum, presubiculum, subiculum, cornu ammonis [CA] 1, CA2/3, CA4, granule cell layer of the dentate gyrus [GC/DG], hippocampal-amygdaloid transition area [HATA], fimbria, molecular layer, hippocampal fissure, hippocampal tail) were calculated according to Iglesias *et al*.^47^. Briefly, this automated analysis of MRI data is based on a construction of a statistical atlas of the hippocampal subfields using ultra-high resolution MRI and then building an algorithm based on Bayesian inference^47^. This atlas was released as part of FreeSurfer (version 6.0) and its applicability and accuracy has been demonstrated^47^, thus offering an alternative to the laborious manual tracing that has been the gold standard for measuring total hippocampal volume in the past and providing a tool that would allow easy replication across laboratories^86^. Segmentation of the hippocampal subfields was visually inspected and all subjects passed the quality control.

All subsequent statistical analyses were done in JMP version 10.0.0 (SAS Institute Inc., Cary, NC). Volumes of left and right hippocampus as well as the subfields were corrected for the total brain volume. Mean scores of the prenatal and early postnatal stressful life events variables had to be log transformed to follow normal distribution. We used two-sided hypothesis tests and significance level of 0.05. With our sample size and power 80% we should have been able to detect medium size or larger effects. First, univariate linear regression assessed associations between early life stress and total left and total right volume of the hippocampus. Potential interactions with sex were assessed. Next, multivariate analysis of variance (MANOVA) assessed associations between the significant predictors of hippocampal volume and volumes of the different subfields in left and right hippocampus. Finally, additional posthoc analyses assessed the potential relationship between hippocampal volume, its predictors, and depressive symptomatology measured by Beck Depression Inventory^79^ (BDI) in young adulthood.

### Data availability

The datasets generated and analysed during the current study are available from the corresponding author upon reasonable request.

## Electronic supplementary material

Supplementary Information
