# Supplementary material for: Perinatal stress and human hippocampal volume: Findings from typically developing young adults
Source: Sci Rep. 2018 Mar 16;8:4696. doi: 10.1038/s41598-018-23046-6 (PMC5856850; doi:10.1038/s41598-018-23046-6)
Supplement: Supplementary file 1 — Supplementary Information [file 41598_2018_23046_MOESM1_ESM.pdf]

TITLE: Perinatal stress and human hippocampal volume: Findings from typically developing young adults

RUNNING TITLE: Perinatal stress and human hippocampal volume

AUTHORS AND AFFILIATIONS

Klára Marečková, PhD[1], Radek Mareček, PhD[2], Petra Bencurova, Mgr[1], Jana Klánová, PhD[4], Ladislav Dušek, PhD[4,5], Milan Brázdil, MD, PhD [1, 3]

[1] Behavioral and Social Neuroscience Research Group, Central European Institute of Technology, Masaryk University (CEITEC MU), Brno, CZ

[2] Multi-modal and Functional Neuroimaging Research Group, Central European Institute of Technology, Masaryk University (CEITEC MU), Brno, CZ

[3]Department of Neurology, St. Anne's University Hospital and Faculty of Medicine, MU, Brno, CZ

[4]Research Centre for Toxic Compounds in the Environment (RECETOX), Faculty of Science, MU, Brno, CZ

[5] Institute of Biostatistics and Analyses (IBA), MU, Brno, CZ

SUPPLEMENTARY METHODS

List of the 40 questions included in the Stressful Life Events questionnaire and answered on a 5-point Likert scale regarding the stressful experience.

1. Death of your partner.
2. Death of one of your children.
3. Death of your friend or relative.
4. One of your children was ill.
5. One of your partners was ill.
6. One of your friends or relatives was ill.
7. You were hospitalized.
8. You had legal issues.
9. You got divorced.
10. You found out your partner does not want your child
11. You were seriously ill.
12. Your partner lost a job.
13. Your partner had problems in his job.
14. You had problems in your job.
15. You lost your job.
16. Your partner broke up with you.
17. Your partner had legal issues.
18. You broke up with your partner.
19. Your income decreased.
20. You argued with your partner.
21. You had an argument with your family or friends.
22. You moved.
23. Your partner hurt you physically.
24. You lost your accommodation.
25. You had serious financial difficulties.
26. You got married.
27. Your partner physically hurt your children.
28. You tried to kill yourself.
29. You were convicted of violating a law.
30. You were bleeding and thought you might lose your child.
31. You started a new job.
32. You were on an examination whether your child might have any defects.
33. You found out your child might not be completely healthy.
34. You found out that something happened that might hurt the health of your child.
35. You tried to abort your pregnancy.
36. You took an exam.
37. Your partner was emotionally cruel to you.
38. Your partner was emotionally cruel to your children.
39. Somebody robbed your apartment/house or a car.
40. You had an injury.

List of the 33 questions included in the Dysregulated Mood and Wellbeing questionnaire and answered on a 4-point Likert scale regarding the frequency of these experiences or feelings in the past month.

1. Do you get angry without a particular reason?
2. Do you suffer from dizziness or difficult breathing?
3. Did you feel like you are going to faint?
4. Do you suffer from digestive problems?
5. Do you feel like life is too difficult?
6. Do you feel nervous and insecure?
7. Do you have tingling sensations in your body, arms or legs?
8. Do you regret most of your past behavior?
9. Do you sometimes experience panic?
10. Do you sometimes have very small or no appetite?
11. Do you sometimes wake up unusually early even though your child did not wake you up?
12. Do you worry a lot?
13. Do you feel tired or exhausted?
14. Do you have long periods of sadness?
15. Do you feel tense?
16. Do you sleep well?
17. Do you sometimes feel like you are going to collapse?
18. Do you often sweat a lot or experience your heartis pounding?
19. Do you need to cry?
20. Do you have bad dreams that make you upset after awakening?
21. Are you losing your ability to empathize with other people?
22. Can you think and decide quickly?
23. Do you need to make an extra effort to cope with difficulties?
24. Are you able to laugh and see life from the good perspective?
25. Were you looking forward to future events?
26. Did you blame yourself unnecessarily when something did not work out?
27. Did you worry or feel anxious without a relevant reason?
28. Were you afraid or panic without a relevant reason?
29. Were you overwhelmed by everything in your life?
30. I was so unhappy I had sleep difficulties.
31. I was sad and miserable.
32. I was so unhappy that I had to cry.
33. I was thinking about hurting myself.

List of the 36 questions included in the Anxiety/co-dependence questionnaire and answered on a 4-point Likert scale regarding the frequency of these thoughts or feelings.

1. I feel insecure when I have to say goodbye.
2. I'm worried about the impressions I make on other people.
3. I avoid saying what I really think because I'm worried to be rejected.
4. I feel insecure when meeting new people.
5. If people knew the real myself they would not like me.
6. I feel secure in an intimate relationship.
7. I don't get angry with people because I'm worried I could harm them.
8. I don't feel well after an argument with a friend until we reconcile.
9. I always care about the feelings of others.
10. I'm anxious not to be criticized for what I've said or done.
11. I always notice when somebody overlooks me.
12. I'm worried to lose somebody close.
13. I think people like me in general.
14. I'd rather do something I don't want to than to offend somebody or make him/her angry.
15. I believe that I did something good only if somebody tells me.
16. I retreat to please somebody who is close to me.
17. I feel anxious when I have to say goodbye.
18. I'm happy if somebody praises me.
19. I'm afraid I'll be overwhelmed by my feelings.
20. I can make other people happy.
21. I found that it's difficult for me to be angry at other people.
22. I'm worried to criticize other people.
23. I don't feel comfortable when somebody criticizes what I do.
24. If people knew the real myself they would have worse opinion about me.
25. I always expect to be criticized.
26. I'm never sure whether others are satisfied with me.
27. I don't like people to get to know me well.
28. I find it hard to calm myself when I get angry.
29. I feel like others don't understand me.
30. I'm worried about what others think of me.
31. I'm not happy when people I know don't admire me.
32. I'm never rude to anyone.
33. I take care not to hurt other people's feelings.
34. It hurts me when somebody is angry at me.
35. I evaluate myself mostly according to what other people think of me.
36. I really care about what other people think of me.

SUPPLEMENTARY RESULTS

Supplementary Table 1: Pairwise correlations between the different measures of prenatal and early postnatal stress (ordered from the highest to the lowest).

Variable	by Variable	Correlation	Signif Prob
Stressful life events during first six months after birth	Stressful life events during first half of pregnancy	0,6795	<,0001
Dysregulated mood and wellbeing at 18 months after birth	Dysregulated mood and wellbeing at six months after birth	0,6584	<,0001
Dysregulated mood and wellbeing at 18 months after birth	Dysregulated mood and wellbeing during first weeks after birth	0,5716	<,0001
Stressful life events during second half of pregnancy	Stressful life events during first half of pregnancy	0,5648	<,0001
Stressful life events during first six months after birth	Stressful life events during second half of pregnancy	0,5503	<,0001
Stressful life events during 6 to 18 months after birth	Dysregulated mood and wellbeing at 18 months after birth	0,5196	<,0001
Dysregulated mood and wellbeing at six months after birth	Dysregulated mood and wellbeing during first weeks after birth	0,5087	<,0001
Stressful life events during second half of pregnancy	Dysregulated mood and wellbeing during first weeks after birth	0,4997	<,0001
Stressful life events during first half of pregnancy	Dysregulated mood and wellbeing at six months after birth	0,4911	<,0001
Stressful life events during first half of pregnancy	Dysregulated mood and wellbeing at 18 months after birth	0,4879	<,0001
Stressful life events during first six months after birth	Dysregulated mood and wellbeing at six months after birth	0,4712	<,0001
Stressful life events during first half of pregnancy	Dysregulated mood and wellbeing during first weeks after birth	0,4624	<,0001
Stressful life events during 6 to 18 months after birth	Stressful life events during first half of pregnancy	0,4453	<,0001
Stressful life events during 6 to 18 months after birth	Stressful life events during first six months after birth	0,4396	<,0001
Stressful life events during 6 to 18 months after birth	Stressful life events during second half of pregnancy	0,4049	<,0001
Stressful life events during first six months after birth	Dysregulated mood and wellbeing at 18 months after birth	0,3855	<,0001
Stressful life events during second half of pregnancy	Dysregulated mood and wellbeing at 18 months after birth	0,3806	<,0001
Stressful life events during first six months after birth	Dysregulated mood and wellbeing during first weeks after birth	0,3728	<,0001
Stressful life events during 6 to 18 months after birth	Dysregulated mood and wellbeing during first weeks after birth	0,3389	0,0003
Anxiety and co-dependence during first weeks after birth	Dysregulated mood and wellbeing during first weeks after birth	0,3255	0,0003
Stressful life events during 6 to 18 months after birth	Dysregulated mood and wellbeing at six months after birth	0,3251	0,0004
Anxiety and co-dependence during first weeks after birth	Dysregulated mood and wellbeing at six months after birth	0,3188	0,0005
Stressful life events during first half of pregnancy	Anxiety and co-dependence during first weeks after birth	0,2368	0,0246
Anxiety and co-dependence during first weeks after birth	Dysregulated mood and wellbeing at 18 months after birth	0,2078	0,0293
Stressful life events during second half of pregnancy	Dysregulated mood and wellbeing at six months after birth	0,1909	0,0392
Stressful life events during second half of pregnancy	Anxiety and co-dependence during first weeks after birth	0,1727	0,0614
Stressful life events during first six months after birth	Anxiety and co-dependence during first weeks after birth	0,1422	0,1261
Stressful life events during 6 to 18 months after birth	Anxiety and co-dependence during first weeks after birth	0,0945	0,3259
Birth weight	Stressful life events during first half of pregnancy	0,0865	0,4099
Birth weight	Dysregulated mood and wellbeing at six months after birth	0,0129	0,8887
Birth weight	Dysregulated mood and wellbeing during first weeks after birth	-0,031	0,7389
Birth weight	Anxiety and co-dependence during first weeks after birth	-0,035	0,7063
Birth weight	Dysregulated mood and wellbeing at 18 months after birth	-0,0409	0,6655
Birth weight	Stressful life events during 6 to 18 months after birth	-0,0415	0,6627
Birth weight	Stressful life events during first six months after birth	-0,1067	0,2462
Birth weight	Stressful life events during second half of pregnancy	-0,1518	0,1009
